# Epigenetics and MicroRNAs in Cancer

**DOI:** 10.3390/ijms19020459

**Published:** 2018-02-03

**Authors:** Alice Ramassone, Sara Pagotto, Angelo Veronese, Rosa Visone

**Affiliations:** 1 Ageing Research Center and Translational Medicine-CeSI-MeT, 66100 Chieti, Italy; alice.ramassone@unich.it (A.R.); sara.pagotto@unich.it (S.P.); 2 Department of Medical, Oral and Biotechnological Sciences, G. d’Annunzio University Chieti-Pescara, 66100 Chieti, Italy

**Keywords:** microRNAs, epigenetics, human cancer

## Abstract

The ability to reprogram the transcriptional circuitry by remodeling the three-dimensional structure of the genome is exploited by cancer cells to promote tumorigenesis. This reprogramming occurs because of hereditable chromatin chemical modifications and the consequent formation of RNA-protein-DNA complexes that represent the principal actors of the epigenetic phenomena. In this regard, the deregulation of a transcribed non-coding RNA may be both cause and consequence of a cancer-related epigenetic alteration. This review summarizes recent findings that implicate microRNAs in the aberrant epigenetic regulation of cancer cells.

## 1. Introduction

In 1942, Conrad Waddington (1905–1975) introduced for the first time the term “epigenetics” in a paper entitled “The Epigenotype,” defining it as “the branch of biology which studies the causal interactions between genes and their products which bring the phenotype into being” [1]. The meaning of this word has gradually evolved since the exponential growth of genetics and in-depth knowledge of this phenomenon. At present, the definition of “epigenetics” as “the study of changes in gene function that are mitotically and/or meiotically heritable and that do not entail a change in DNA sequence” is generally accepted [2,3,4,5].

The most common mammalian epigenetic modifications are (i) DNA methylation at the 5-carbon of the cytosine and (ii) histone acetylation and methylation [6,7]. However, it has become evident that (iii) non-coding RNAs have an important role in the molecular mechanisms that sustain epigenetics [8]. Alterations of these factors can cause abnormal epigenetic patterns at canonical promoter boxes or distant regulatory elements and may contribute to deregulate critical genes involved in proliferation, programmed cell death, and cell differentiation [9,10,11].

The initiation and progression of human cancer is thought to be driven by combinations of epigenetic and genetic alterations that activate multistep programs of carcinogenesis [12,13]. Recent evidence shows that epigenetic reprogramming of cancer stem cell (CSC) is a key step in the earliest phases of neoplastic progression. This promotes the clonal expansion of aberrant cells prone to subsequent genetic and epigenetic alterations associated with neoplastic evolution [13,14,15].

Compared to aberrant DNA methylation, little is known about abnormal histone modifications in carcinogenesis, but this is an area of great interest given its importance for chromosome remodeling and, therefore, for transcription regulation, DNA repair, chromosome condensation, and segregation [16,17,18,19,20,21]. Non-coding RNAs can be distinguished in long non-coding RNAs (lncRNAs) and small RNAs including microRNAs, focus of this review. While a role as new epigenetic factors has been assigned to lncRNAs [22,23], microRNAs need a more in-depth discussion.

MicroRNAs (miRNAs or miRs) are small, noncoding RNAs that directly modulate gene expression at the post-transcriptional level binding predominantly to 3′-untranslated region (3′UTR) of target messenger RNAs (mRNAs) in a sequence-specific manner [24,25].

Through this regulation, miRNAs play a pivotal role in several cellular processes, including proliferation, cell cycle control, programmed cell death, differentiation, invasiveness, and tissue specific functions such as immune responses, hormone secretions, and angiogenesis. All these processes are implicated in the development and evolution of cancer [26,27,28,29]. Genome-wide analysis has demonstrated that miRNAs expression is deregulated in most cancer types through various mechanisms, including defects in the miRNA biogenesis machinery, amplification/deletion of the region encompassing the miRNA, or aberrant transcriptional control [26]. Compelling evidence demonstrated that miRNAs can also be deregulated in cancer by abnormal CpGs methylation and/or histone modifications [30]. On the other hand, several miRNAs are not only regulated by epigenetic mechanisms, but themselves have an active role on the epigenetic machinery, creating highly-controlled feedback circuits that finely tune gene expression. These subgroups of miRNAs, called “epi-miRNAs”, are often deregulated in human cancer and target specific epigenetic regulators, such as components of the polycomb repressive complexes 1 and 2 (PRC1 and PRC2), DNA methyl-transferases (DNMTs) and histone deacetylases (HDACs) enzymes, and the Retinoblastoma-Like protein 2 (RBL2) [31,32,33,34,35,36]. Moreover, it was shown that miRNAs are also present in the nucleus [37,38], where they regulate gene expression via distinct mechanisms.

This review summarizes the state-of-the-art of an intimate but still largely unknown networking between epigenetics and microRNAs in human cancer.

## 2. Epigenetic Alterations of miRNAs in Cancer

### 2.1. By DNA Methylation

DNA methylation occurs in vertebrate cells at carbon-5 of the cytosine ring in CpG di-nucleotides. The reaction is catalyzed by DNMTs using S-adenosyl-methionine as methyl-donor. It is a normal process used by cells to maintain the physiological expression of genes and to maintain mono-allelic expression of imprinted genes [39]. About 70% of the promoters in the human genome are associated with regions characterized by a high frequency of CpGs (CpG islands, CGIs) that can be methylated by the DNA methylation machinery [40]. In 2007, Weber et al. found that 155 out of 332 human miRNA investigated (47%) were associated with CGIs, suggesting that miRNAs were subject to transcriptional regulation by DNA methylation [41].

The first evidence of regulation of miRNAs by DNA methylation came from a profiling of miRNA expression of the T24 bladder cancer cell line after treatment with the DNA de-methylating agent 5-Aza-2′-deoxycytidine (5-AZA), in combination with an HDAC inhibitor (4-phenylbutyric acid; 4-PBA). Seventeen out of 313 miRNAs were deregulated after treatment. Among these, *miR-127* was up-regulated, with consequent down-regulation of its target, the proto-oncogene B-cell lymphoma 6 (BCL6) [42].

In another study, after stable depletion of *DNMT1* and *DNMT3B* in the HCT116 colorectal cancer cell line, the *miR-124a*, *miR-373*, and *miR-517c* were demonstrated to be transcriptionally inactivated by CGI methylation [43]. The same authors also found a signature of microRNA hyper-methylated in metastatic cell lines from colon (SW620), melanoma (IGR37) and head and neck (SIHN-011B) cancers. Hyper-methylation-associated silencing of *miR-9*, *miR-34b/c*, and *miR-148a* observed in those metastatic cell lines was also evident in primary colon, breast, lung, head, and neck carcinomas and melanomas [44].

After these general approaches to identify miRNAs aberrantly expressed by DNA methylation in cancer cells [41,42,43], several tumor specific studies were performed to obtain exploitable data in cancer research.

*MiR-9*, *miR-34b/c*, *miR-124a*, and *miR-148a* hyper-methylation was confirmed in breast cancer cells [45,46,47], together with *let-7a*, *miR-10b*, *miR-125b*, *miR126*, *miR-152*, *miR-195/497*, *miR-200* family, and miRs at the imprinted locus *DLK1-DIO3* region [48,49,50,51,52,53,54,55,56]. Moreover, down-regulation by methylation of the *miR-149* was reported in clinical cases of chemoresistant breast cancer [57].

In pancreatic ductal adenocarcinoma (PDAC) were found hyper-methylated the *miR-9-1*, *miR-124*s, *miR-192*, *miR-615-5p*, and *miR-1247*, suggesting tumor suppressor roles in this type of cancer [58,59,60,61,62]. Differently from breast and other cancers, *miR-200a* and *miR-200b* were reported to be expressed and de-methylated in PDAC [63].

In gastric cancer (GC) cell lines and in about 70% of primary GCs the *miR-34b/c* and the *miR-181c* genes were found to be epigenetically silenced by CGI hyper-methylation [64]. This was postulated to contribute to the activation of notch 4 (NOTCH4) and KRAS proto-oncogene, GTPase (KRAS), targets of these miRs [65]. Aberrant methylation of the *miR-1*, *miR-9*, *miR-129*, *miR-10a/b*, of the *miR-200a/b/429* locus, and of *miR-*33b was observed in GC [66,67,68,69,70,71,72]. Of note is the analysis of the methylation status of *miR-124* in the normal gastric mucosa of GC patients and healthy volunteers with or without *Helicobacter pylori* infection. Among the healthy volunteers, the cases with *H. pylori* infection showed higher levels of methylation of *miR-124* than in samples without infection, and among the non-infected samples, gastric mucosa from gastric cancer patients show higher levels of methylation of *miR-124* than in the mucosa from healthy donors. These data suggest that the aberrant methylation of *miR-124* is an early event in the pathogenesis of GC [73].

In hepatocellular carcinoma (HCC), several miRs were confirmed to be aberrantly methylated such as *miR-1*, *miR-9*, *miR-34b*, *miR-124*, *miR-148a* and, *miR-200b* [74,75,76,77,78]. A microRNA host gene involved in HCC, the insulin like growth factor 2 (*IGF2*), shows hyper-methylation of 3 CpGs at the intron 2, immediately upstream the *miR-483*, associated with strong expression of this miR. When methylated, those CpGs cannot bind the transcriptional repressor CCCTC-binding factor (CTCF), permitting microRNA transcription [79]. In the same tumor type, *miR-221* is up-regulated [80]. Hypo-methylation of the region upstream *miR-221* in a cellular context holding the wild type tumor protein p53 (TP53) seems to enable its expression [81]. A recent study shows a global, cancer-specific microRNA cluster hypo-methylation in HCCs that do not harbor hepatitis C virus (HCV) or hepatitis B virus (HBV) infections [82].

Aberrant methylation of several miRs is a recurrent theme in cancer, which underlines their biological importance in general tumorigenic processes. The *miR-*9 has been reported aberrantly methylated in ovarian, renal, liver, lung, colorectal cancer, and multiple myeloma. Its silencing allows up-regulation of important oncogenic products, such as cyclin G1 (CCNG1) and epidermal growth factor (EGF) [83]. *miR-34*s are similarly methylated in several type of cancers, and their silencing affects cellular stemness by targeting CD44 molecule (CD44) and notch 1 (NOTCH1), cell cycle by targeting MYC proto-oncogene, bHLH transcription factor (MYC) and cyclin dependent kinase 6 (CDK6), and apoptosis by targeting BCL2 apoptosis regulator (BCL2) protein[84,85,86,87,88]. Of note is *miR-124*, whose expression was found to be deregulated by hyper-methylation in 14 different tumor types (Table 1). *MiR-124* targets four lncRNAs (metastasis associated lung adenocarcinoma transcript 1 (*MALAT1*); HOX transcript antisense RNA (*HOTAIR*); *HOXA11 antisense RNA* (*HOXA11-AS*) and long intergenic non-protein coding RNA, regulator of reprogramming (*LINC-ROR*)) [89,90,91,92] that act as sponges for the miRs, as the *miR-124*, inhibiting its oncosuppressor functions [93,94,95,96]. *MiR-137* was also hyper-methylated in nine different tumor types, which is consistent with the fact that this microRNA controls many cellular processes deregulated in cancer, such as cell cycle progression by targeting CDK6 [97], tumor glutamine metabolism by targeting solute carrier family 1 (neutral amino acid transporter), member 5 (ASCT2) [98], and chromosome remodeling by targeting the enhancer of zeste 2 polycomb repressive complex 2 subunit (EZH2) [99].

*MiR-200a/b-429* and *miR-200c-141* play a pivotal role in the epithelial to mesenchymal transition (EMT) by targeting the transcription factors zinc finger E-box binding homeobox 1 and 2 (ZEB1; ZEB2) [100,101,102,103], and in cell proliferation by targeting phosphatase and tensin homolog (PTEN) and KRAS [104,105]. These targets play a role also in cellular stemness. Indeed, the stem-like cell fractions isolated from metastatic breast cancers displayed loss of *miR-200*. Moreover, it has been demonstrated that in the stem-like phenotype, the *miR-200c-141* cluster was repressed by promoter CpG hyper-methylation, whereas the *miR-200b*-*200a*-*429* cluster was silenced through polycomb group-mediated histone modifications [106].

### 2.2. By Histone Modifications

Histone post-translational modifications include methylation, phosphorylation, acetylation, ubiquitination, and sumoylation. Histone methylation and histone acetylation are covalent post-translational modifications by which methyl or acetyl groups are transferred to amino acids on the histone tails, modifying gene accessibility and hence expression by alteration of the chromatin structure. Specifically, acetylation is associated with an open chromatin state marking active region of transcription, while methylation can be present both in actively transcribed and in repressed regions [107].

The first evidence of deregulation of miRNA due to histone modification in cancer cells was reported by Scott et al. in 2006. These authors demonstrated the aberrant expression of 27 miRNAs after treatment of SKBr3 breast cancer cells with an HDACs inhibitor [108]. In chronic lymphocytic leukemia (CLL) and mantle cell lymphoma (MCL), *miR-15a* and *miR-16* are epigenetically silenced due to overexpression of HDACs. Indeed, treatment with a deacetylase inhibitor restored the expression of these miRNAs in CLL cells, with associated down-regulation of MCL-1 levels and decreased CLL cell survival [109,110]. In 2006, Mertens et al. demonstrated that genes at the 13q14.3 region, which harbors *miR-15a* and *miR-16-1*, shows mono-allelic expression in B-CLL cells independently of the chromosome copy number. Mono-allelic expression was due to different chromatin packaging of the two copies of 13q14.3; indeed, treatment with 5-aza-CdR or trichostatin A (TSA) induced bi-allelic expression at 13q14.3 [111]. In line with these evidences, we have recently found in CLL a double allele-specific transcriptional regulation of the *miR-15a/16-1* cluster involving both the RNA polymerase II and the RNA polymerase III. If either the epigenetic silencing of the 13q14.3 region or the 13q14 deletion affects the allele transcribed by the RNA polymerase II, the allele transcribed by the RNA polymerase III can be un-masked [112]. The oncogenic *miR-155* has been found to be epigenetically repressed in breast cancer by BRCA1, DNA repair associated (BRCA1), which recruits HDAC2 on the miR-155 promoter. *MiR-155* is up-regulated only in breast cancer cells with loss of wild-type BRCA1 or mutant-BRCA1, since HDCA2 cannot be recruited on the miR promoter [113]. Recent evidence indicates that in prostate cancer, the mocetinostat, a class I selective inhibitor of the HDACs, up-regulates *miR-31* with consequent loss of expression of its target E2F transcription factor 6 (E2F6), induction of apoptosis, and reduction in cancer growth [114]. *MiR-449* was repressed by HDAC1-3 in HCC cell line [115].

Wang et al. in 2012 demonstrated in HCC that HDAC1 and HDAC3 act as negative regulators of *miR-224* expression, whereas the histone acetyl-transferase EP300 is a positive regulator. They suggest that in normal cells, the *miR-224* locus is maintained transcriptionally quiescent by HDAC1 and HDAC3, while during cellular transformation, *miR-224* expression is activated by overexpression of EP300. Finally, they propose that EP300 could represent a potential drug target to reverse *miR-224* overexpression in HCC patients [116].

In 2009, Yang et al. demonstrated that *miR-449a/b* expression in an osteosarcoma cell line was epigenetically repressed through tri-methylation of the lysine 27 on the histone H3 (H3K27me3), reversible by epigenetic drug treatment [117]. Multiple miRNAs are down-regulated in HCC by EZH2, which mediates H3K27me3, such as *miR-139-5p*, *miR-125b*, *miR-101*, *let-7c*, and *miR-200b* [118]. In prostate cancer, *miR-181a*, *miR-181b*, *miR-200b*, *miR-200c*, and *miR-203* were found epigenetically repressed by EZH2 [119]. Recently, *miR-31* was also identified to be repressed by EZH2 in prostate cancer [120].

MicroRNAs epigenetically regulated in cancer are reported in Table 1.

## 3. MiRNAs as Epigenetic Regulators

**Figure 1:**
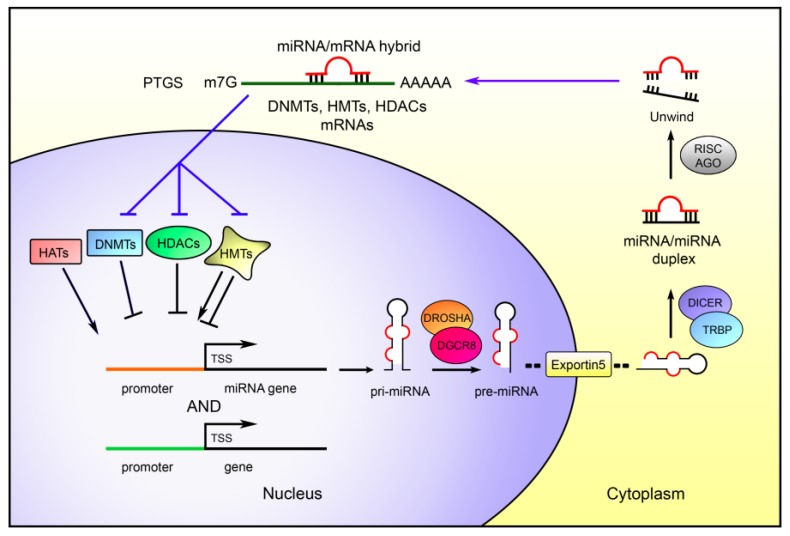
**Feedback circuit between microRNAs and epigenetic machinery**. The epigenetic modification, such as promoter CpG island hyper- or hypo-methylation and/or histone modifications, affect miRNAs and genes transcription. MiRNAs can themselves regulate the epigenetic machinery by post-transcriptional gene silencing (PTGS), targeting DNMTs, HDACs, and the histone methyl-transferases (HMTs), establishing epigenetic pathway loops. In the figure, black lines represent the pathway starting from the epigenetic modifications and ending with the miRNAs maturation, while blue lines represent the pathway from the mature miRNA to the post transcriptional gene silencing of the epigenetic machinery.

**Figure 2:**
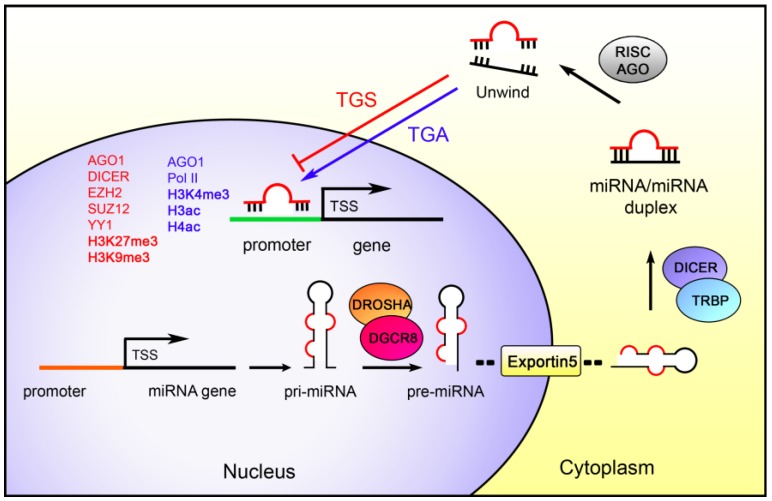
**MicroRNAs regulate gene transcription**. Nuclear miRNAs can mediate both transcriptional gene silencing (TGS) and transcriptional gene activation (TGA) by targeting gene promoters. During the TGS, AGO1, DICER, EZH2, SUZ12, and YY1 proteins can be recruited on target promoters to induce the silencing through enrichment of H3K9me3 and H3K27me3. Instead, during the TGA, target promoters exhibit the enrichment of the RNA polymerase II, H3K4me3, and H3ac, H4ac; moreover, AGO1 was also found to be associated to target promoters during TGA. In the figure, black arrows indicate the miRNAs biogenesis pathway, and red and blue lines represent miRNAs translocated back to the nucleus to mediate TGS or TGA, respectively. Chromatin modifications are represented in bold.

Although miRNAs are mitotically and meiotically hereditable factors [222,223,224] able to regulate gene expression without involving changes in the DNA sequence, their classification as epigenetic factors is still debated [225]. However, growing evidence shows their substantial role in the control of several canonical epigenetic mechanisms. Specifically, miRNAs regulate at the post-transcriptional level many epigenetic-related-genes (Figure 1). Nevertheless, miRNAs can also act in the nucleus by stimulating or repressing genes transcription in a manner strictly correlated to the chromatin state (Figure 2).

### 3.1. Post-Transcriptional Gene Silencing by miRNAs

MiRNAs regulate at the post-transcriptional level several epigenetic factors involved in transcriptional regulation, such as DNMTs, PRC1 and PRC2, heterochromatin protein 1 (HP1), and HDACs. Deregulation of these proteins induced by aberrant expression of miRNAs could lead to the epigenetic silencing of tumor suppressor genes, believed to be an early driver of oncogenesis [226].

Deregulation of DNMTs was observed in cancer [227]. The *miR-29* family, down-regulated in lung cancer, targets DNA methyl-transferase 3 alpha and 3 beta (DNMT3A-B) [31]. Exogenous expression of *miR-29s* results in a decrease of global DNA methylation and in the re-expression of tumor suppressor genes in lung cancer and in acute myeloid leukemia [31,32]. Moreover, in hepatocellular carcinoma, *miR-29a* modulates both the DNA methyl-transferase 1 (DNMT1) and DNMT3B [228]. A DNMT3B splice variant is regulated by *miR-148* through the binding to the coding region in cancer cell lines [229]. In cholangiocarcinoma, *miR-148*a and *miR-152* target DNMT1; reduced expression of these miRNAs contributes to increased DNMT1 activity, which affects transcription of the tumor suppressor genes Ras association domain family member 1 (*RASSF1A*) and cyclin-dependent kinase inhibitor 2A (*p16INK4a*) [34].

The DNMT family was also found to be regulated by *miR-K12-4-5p*, which is encoded by Kaposi’s sarcoma-associated herpesvirus (KSHV). *miR-K12-4-5p* directly down-regulates RBL2, a repressor of *DNMT3A-B* mRNA transcription [230]. Thus, enforced expression of this viral miRNA reduces RBL2 protein level and increases *DNMT1* and *DNMT3A-B* mRNA levels, leading to global hypo-methylation [33].

PRC2, one of the two classes of Polycomb group proteins was found to cooperate with DNMTs in silencing of target genes [231]. PRC2 mediates the di- and tri-methylation of H3K27 (H3K27me2 and H3K27me3) through the SUZ12 polycomb repressive complex 2 subunit (SUZ12) and EZH2 [232,233], each of which is regulated by miRNAs. For instance, *miR-200b* negatively regulates the expression of SUZ12 in breast cancer stem cells (BCSC). Loss of *miR-200b* results in an increase of SUZ12 binding at the E-cadherin (*CDH1*) promoter, leading to the aberrant H3K27me3 and *CDH1* repression. The pathway involving *miR-200b*, SUZ12, and the *CDH1* is important for BCSC growth: induced expression of *miR-200b* or SUZ12 silencing block tumor formation in in vivo models [234]. In glioma stem-like cells, a tumor subpopulation with self-renewal capacity, down-regulation of SUZ12 depends on *miR-128* expression. The restoration of *miR-128* affects SUZ12 levels and reduces cell proliferation [235].

EZH2, another member of the PRC2 complex, is over-expressed in cancer, enhancing cell growth and transformation [236,237]. It was found to be regulated by *miR-26a* and *miR-101*. *miR-26a* influences cell cycle progression in Burkitt’ lymphoma cell lines by targeting EZH2 [238], while *miR-101* attenuates cell proliferation in bladder transitional carcinoma and prostate cancer cell lines [239,240]. 

A stable gene silencing is maintained by PRC1, which recognizes H3K27me3, catalyses histone H2A ubiquitylation, and promotes chromatin compactation [241]. It contains several subunits, among which is BMI1 proto-oncogene, polycomb ring finger (BMI1). BMI1 is up-regulated in cancer and promotes stem cell self-renewal [242]. BMI1 expression is controlled by different miRNAs in cancer. In glioma, the *miR-128* targets BMI1 leading to reduced self-renewal capacity [243]. In ovarian cancer, BMI1 is regulated by *miR-15a* and *miR-16*-*1* and induced expression of these miRNAs decreases BMI1 protein levels, reducing ovarian cancer cell proliferation [244]. In endometrial cancer cells, *miR-194* negatively regulates BMI1 and reduces cell invasion [245]. By targeting BMI1, *miR-218* affects the migration, invasion, and proliferation of glioma cells and blocks self-renewal ability [246]. In multiple myeloma, *miR-203* is down-regulated, and its restoration suppresses BMI1 expression and inhibits myeloma cell growth [247].

HDACs interact with PRC2 [248] and are up-regulated in various type of cancer [249]. *miR-449a* is down-regulated in prostate cancer and its expression negatively correlates with the expression of its direct target, the histone deacetylase 1 (HDAC1); introduction of *miR-449a* in prostate cancer cells affects cell growth and viability, in part by targeting HDAC1 [250]. However, in different cancer cell models, HDAC1 was demonstrated to act as a repressor of this miR, suggesting a loop that regulates the expression of these genes [115]. In hepatocellular carcinoma, *miR-145* is down-regulated and negatively regulates the histone deacetylase 2 (HDAC2) expression. Overexpression of *miR-145* reduces the tumorigenic potential of hepatocellular carcinoma cells in vitro and in vivo, recapitulating the effects of HDAC2 inhibition [251]. In B-lymphoma cells the histone deacetylase 4 (HDAC4) is down-regulated by *miR-155.* In this context, HDAC4 acts as tumor suppressor, reducing proliferation and promoting apoptosis [252].

The HP1 family is involved in several functions, including heterochromatin spread and chromatin condensation [253]. The HP1 family is deregulated in cancer [254]. In colorectal cancer, the HP1γ protein encoded by chromobox 3 gene (*CBX3*), is overexpressed and associated with poor prognosis, while *miR-30a* is down-regulated. It was demonstrated that *miR-30a* targets HP1γ in colon cancer cells inhibiting cell growth and tumour progression in vitro and in vivo [255].

Epigenetic protein factors targeted by miRNAs are shown in Table 2.

### 3.2. miRNAs Regulate Gene Transcription

Several miRNAs were identified in the nuclear compartment [38]. *miR-29b*, which is localized in the nucleus, shows in the 3′end a hexanucleotide motif that drives nuclear localization [265]. In this, compartment, miRNAs act on gene promoters, both activating and repressing gene expression (Table 3). Interestingly, the argonaute 1, RISC catalytic component (AGO1), which interacts with miRNAs, was also found to drive transcriptional gene silencing in the nucleus [266,267] or to bind and cooperate with RNA Polymerase II on actively transcribed promoters [268].

#### 3.2.1. MiRNAs Transcriptional Gene Silencing (TGS)

The TGS mechanism mediated by small RNAs was identified in human cells [277]; it involves both AGO1-2 and small interfering RNAs that recognize the target promoter region by sequence complementarity [266,267]. Furthermore, the target region exhibits chromatin markers associated with an inactive state, such as methylation of lysines 27 and 9 of histone H3 (H3K27 and H3K9) [266,278]. Recent studies demonstrated that miRNAs could influence the expression of target genes with similar mechanisms.

*MiR-320* was the first identified miRNA able to repress gene transcription. It is located within the RNA polymerase III subunit D (*POLR3D*) promoter region in antisense orientation. It acts as *cis*-regulatory element for transcriptional silencing of the *POLR3D* gene by recruiting AGO1 and EZH2 and causing tri-methylation of the H3K27 on the *POLR3D* promoter [272]. This epigenetic mechanism could be relevant in cancer since the POLR3D gene product is a component of the RNA polymerase III, whose abnormal activity is characteristic of cancer cells [279].

*MiR-10a* recognizes a complementary region within the homeobox D4 (*HOXD4*) promoter and reduces *HOXD4* gene expression in breast cancer cells. This mechanism requires the presence of the dicer 1, ribonuclease III protein (DICER) and AGO1-3 and is accompanied by tri-methylation of H3K27 and de novo DNA methylation at target regions [269]. In breast cancer cells, overexpression of a synthetic *miR-423-5p* inhibits the expression of the Progesterone Receptor (*PGR*) gene, a prognostic marker of breast cancer [280], by reducing RNA polymerase II binding and enriching silent chromatin markers on *PGR* gene promoter [274]. In patients with acute myeloid leukemia, *miR-223* expression shows an inverse correlation with the expression of *NFI-A*, a transcription factor whose expression impacts on erythroid or granulocytic lineage commitment [281]. During granulopoiesis induced by retinoic acid, *miR-223* represses transcription of nuclear factor I A (*NFI-A*) by recruiting DICER and the Polycomb group proteins YY1 transcription factor (YY1) and SUZ12 on its promoter to induce a silent chromatin state with the increase of H3K27me3 [271].

#### 3.2.2. MiRNAs Transcriptional Gene Activation (TGA)

MiRNAs are also able to induce gene expression by activating the target gene promoter. This is accompanied by an active chromatin state that includes an increase of di-methylation and tri-methylation of histone H3K4 (H3K4me2 and H3K4me3) and acetylation of histone H3 and H4 (H3ac and H4ac) [282]. *MiR-373* is the first discovered miRNA involved in the TGA. In prostate cancer cells, it induces the expression of the tumor suppressor gene *CDH1* by complementary binding to its promoter with consequent enrichment of RNA polymerase II on the target promoter [273]. *MiR-205* is down-regulated in prostate cancer, and its restoration reduces cell proliferation by activating the interleukin 24 and interleukin 32 (*IL24* and *IL32*) genes. Indeed, *miR-205* induces expression of *IL24* and *IL32* by targeting their promoters, thus leading to an enrichment of RNA polymerase II and of H3ac, H4ac, and H3K4me2 [270]. The *miR-483* is encoded within an intron of the *IGF2* gene, and overexpression of both *IGF2* and *miR-483* was observed in Wilms’ tumor [275,283]. *MiR-*483 up-regulates *IGF2* transcription by interacting with the 5′UTR of the transcript and by enhancing the interaction with the RNA helicase DExH-Box Helicase 9 (DHX9) [275], a transcriptional co-activator [284]. The cytochrome c oxidase II (*COX2*) is a pro-inflammatory gene that shows two complementary sequences for the *miR-589* on its promoter: by using an anti-*miR-589-5p* in lung cancer cells, a reduction of the basal expression of *COX2* was observed, while enforced expression of *miR-589* results in an increased COX2 protein level [260].

Transcriptional gene activation mediated by miRNAs was also observed in mice: *miR-774* and *miR-1186* binding sites were identified in the promoter of the cyclin B1 (*Ccnb1*). The *miR-774* recruits AGO1 and promotes the enrichment of the RNA Polymerase II and of the histone H3K4 tri-methylation on *Ccnb1* promoter in prostate adenocarcinoma cells [276].

## 4. Others

With the non-coding RNA world, other areas of research involving the epigenetic phenomena are growing. Recently, the findings of ribonucleoside modifications at RNA-expressed sequences (epi-transcriptome) [285,286] opened a new field of research in cancer biology. Those changes can affect microRNAs maturation influencing expression and downstream targets. A modification able to affect microRNAs processing is methylation of the ribonucleoside adenine (N6-methyladenosine, m^6^A): the methylated *pri-let-7e* was processed in *pre-let-7e* more efficiently than the un-methylated *pri-let-7e* [287]. Then, it was shown that Adenosine (A) to Inosine (I) editing on *miR-200b* RNA influences the downstream targeting of the microRNA and, more importantly, correlates with cancer patient prognosis [288]. 

Another field of research that should be explored is the microRNA targeting the non-coding RNAs involved in chromatin remodeling. It was shown that lncRNAs as H19, imprinted maternally expressed transcript (non-protein coding) (*H19*) and *HOTAIR* can act as decoy for microRNAs [89,289,290,291,292], however they also affect chromosome state by binding the epigenetic complex PRC2 [290,293]. It could be possible that the lncRNA-miRNA complexes, other than work as miRNAs decoys, have a functional role in the chromosome remodeling.

## 5. Conclusions

This review underlines the importance of microRNAs in the complex regulatory mechanisms that control cancer epigenetics. MicroRNAs are tightly regulated by epigenetic modifications such as DNA methylation and histone modifications. However, microRNAs themselves strictly regulate the epigenetic machinery at the post-transcriptional level by establishing epigenetic pathway loops. For instance, overexpression of DNMT1 causes hyper-methylation of *miR-148a* that, in turn, targets *DNMT1* [34,52,261].

As reported, microRNAs can also modulate transcription by binding the promoter of target genes, functioning as a scaffold for chromatin modifiers and transcriptional regulators. The finely-tuned epigenetic network that is unveiling highlights a new level of complexity in the regulation mediated by microRNAs, which modulate at several levels the cellular transcriptome.

Epigenetics changing are reversible, and RNAs are targetable. The possibilities to find useful therapeutic targets in the cancer treatment will increase with future research progress in this area.

## Figures and Tables

**Table 1:** Epigenetically regulated miRNAs in human cancer.

*miRNA*	Cancer Type	Epigenetic Modification	Target	Reference
*miR-1*	Hepatocellular, liver, colorectal, lung	DM_hyper_	FOXP1, MET, HDAC4, Pim1	[74,121,122]
*miR-9*	Breast, ovarian, pancreatic, multiple myeloma, renal, gastric, hepatocellular, colorectal, melanoma, head and neck, multiple myeloma, lung	DM_hyper_	CCNG1, IL-6, AP3B1, TC10, ONECUT2, IGF2BP1, MYO1D, ANXA2	[44,47,58,67,75,123,124,125]
*miR-10a*	Gastric, bladder, hepatocellular	DM_hyper_	HOXA1	[69,126,127]
*miR-10b*	Gastric, hepatocellular	DM_hyper_		[70,127]
*miR-15a/16*	Chronic lymphocytic leukemia, mantle cell lymphoma	HDA	BCL2, MCL1	[109,110]
*miR-17-92*	Colorectal	HDA	PTEN, BCL2L11, CDKN1A	[128]
*miR-21*	Ovarian, prostate, colorectal	DM_hypo_, DM_hyper_, HMT	ITGB4	[129,130,131]
*miR-23a-27a*	Hepatocellular	DM_hypo_		[78]
*miR-24*	Nasopharyngeal	DM_hyper_		[132]
*miR-29a/b*	B-cell Lymphoma, chronic lymphocytic leukemia, acute myeloid leukemia, lung	HMT, HDA	MCL1, DNMT3A-B	[31,32,109,133,134]
*miR-31*	Melanoma, prostate, breast	HMT, DM_hyper_, HDA	SRC, RAB27A, MAP3K14, MET, E2F1, E2F2, EXO1, FOXM1, MCM2, E2F6, BMI-1	[120,135,136,137,138,139]
*miR-33b*	Gastric	DM_hyper_		[72]
*miR-34a*	Lung, breast, colon, kidney, bladder, pancreatic cancer cells, melanoma	DM_hyper_	CDK6	[76,140,141]
*miR-34b/c*	Gastric, ovarian, lung, colon, melanoma, head and neck, breast, non-small cell lung, neuroblastoma, hepatocellular, pleural mesothelioma, oral	DM_hyper_	MYC, CDK6, E2F3	[45,68,76,141,142,143,144,145]
*miR-101*	Hepatocellular	HMT		[118]
*miR-106b-25-93 *	Hepatocellular	DM_hypo_		[78]
*miR-107*	Pancreatic	DM_hyper_	CDK6	[146]
*miR-124*	Colon, gastric, hematological, cervical, glioblastoma cells, breast, prostate, neuroblastoma, pancreatic, colorectal, non-small cell lung, acute lymphoblastic leukemia, hepatocellular, renal	DM_hyper_	BCL2, CDK6, VIM, SMYD3, IQGAP1, RAC1	[45,59,73,77,142,144,147,148,149,150,151,152]
*miR-125b*	Breast, hepatocellular	HMT, DM_hyper_	PGF	[45,118,153]
*miR-126*	Bladder, malignant pleural mesothelioma, colorectal, non-small cell lung	DM_hyper_, HDA	VEGF	[154,155,156,157]
*miR-127*	Prostate, bladder, colon, breast, clear cell renal cell carcinoma	DM_hyper_, HDA	DAPK1, BCL6	[42,45,158]
*miR-129*	Gastric, endometrial, colorectal, hepatocellular, hematological	DM_hyper_	SOX4	[68,159,160,161,162,163]
*miR-132*	Pancreas, prostate, breast	DM_hyper_, HDA	TALIN2, HB-EGF	[45,164,165]
*miR-133b*	Colorectal	DM_hyper_		[166]
*miR-137*	Head and neck squamous cells, colorectal, glioblastoma cells, prostate, multiple myeloma, gastric, oral, hepatocellular cells	DM_hyper_	CDK6, E2F6, LSD-1, ASCT2, AURKA	[98,145,151,167,168,169,170,171]
*miR-139*	Hepatocellular, non-small cell lung	HMT	ROCK2	[172,173]
*miR-141*	Clear cell renal cell carcinoma	DM_hyper_, HDA	TET1, TET3, ZEB1	[158,174]
*miR-143*	Leukemia	DM_hyper_	MLL-AF4	[175]
*miR-145*	Prostate, lung adenocarcinoma, non-small cell carcinoma, clear cell renal cell carcinoma	DM_hyper_, HDA	TNFSF10, MUCIN1	[158,176,177,178,179]
*miR-148a*	Colorectal, melanoma, head and neck, breast, pancreas, hepatocellular	DM_hyper_	TGIF2	[44,78]
*miR-149*	Breast	DM_hyper_	NDST1	[57]
*miR-152*	Endometrium, bladder cancer cells, prostate, breast cancer cells	DM_hyper_	DNMT1, E2F3, MET, RICTOR	[34,126,171,180,181]
*miR-155*	Breast, prostate	HDA, DM_hyper_		[113,171]
*miR-181a/b*	Prostate	HMT, DM_hyper_, HDA	RING2	[119]
*miR-181c*	Gastric, prostate, glioblastoma cells	DM_hyper_	NOTCH4, KRAS, NOTCH2	[65,182]
*miR-191*	Breast, hepatocellular	DM_hypo_	TIMP3	[45,183]
*miR-192*	Pancreatic ductal adenocarcinoma	DM_hyper_	SERPINE1	[61]
*miR-193a*	Hepatocellular, acute myeloid leukemia, bladder, breast, oral	DM_hyper_, DM_hypo_	E2F6, SRSF2, PLAU, HIC2	[45,145,184,185,186]
*miR-193b*	Prostate	DM_hyper_, HDA		[187,188]
*miR-195/497*	Hepatocellular	DM_hyper_		[78]
*miR-196b*	Gastric, prostate, hepatocellular	DM_hyper_, DM_hypo_		[127,131,189]
*miR-199a*	Testicular, ovarian	DM_hyper_	PODXL, DDR1	[190,191]
*miR-200a/b/429*	Hepatocellular, prostate, gastric, glioblastoma, pancreatic, bladder	HMT, DM_hyper_, HDA, DM_hypo_	BMI1, RING2	[63,71,118,119,126,192]
*miR-200c/141*	Colon, breast, lung, prostate, non-small cell lung	HMT, DM_hyper_, HDA	DNMT3A TET1, TET3, BMI1, RING2, SOX2, ZEB1, DNMT3A	[119,174,193,194,195]
*miR-203*	Hematological, hepatocellular, endometrial, ovarian, prostate, oral	DM_hyper_, DM_hypo_, HMT, HDA	ABCE1, BMI1, SOX4	[77,119,129,145,196,197]
*miR-205*	Bladder, prostate, ovarian	DM_hypo_, DM_hyper_	BCL2L2	[129,131,139,198]
*miR-218*	Oral squamous cell carcinoma	DM_hyper_	RICTOR	[199]
*miR-219a*	Gastric, endometrial	DM_hyper_		[196,200]
*miR-221*	Hepatocellular	DM_hypo_	MDM2	[81]
*miR-224*	Hepatocellular	HDA, HAT		[116]
*miR-335*	Breast, hepatocellular, gastric	DM_hyper_	RASA1, CRKL	[201,202,203,204]
*miR-342*	Colorectal	DM_hyper_		[205]
*miR-345*	Colorectal	DM_hyper_	BAG3	[206]
*miR-370*	Cholangiocarcinoma, oral squamous cells	DM_hyper_	IRS1	[207,208]
*miR-373*	Cholangiocarcinoma	DM_hyper_, HDA		[209]
*miR-375*	Esophagus, melanoma, prostate, hepatocellular, breast	DM_hyper_	RASFF1(A), PDK1	[45,78,210,211,212]
*miR-376c*	Cholangiocarcinoma	DM_hyper_		[207]
*miR-378*	Hepatocellular	DM_hyper_		[78]
*miR-449a/b*	Osteosarcoma cell line, breast cell line, hepatocellular	HMT, HDA	CDK6, CDC25A, C-MET	[115,117]
*miR-512*	Gastric	DM_hyper_, HDA		[42]
*miR-514*	Clear cell renal cell carcinoma	DM_hyper_, HDA		[158]
*miR-585*	Oral squamous cell carcinoma	DM_hyper_		[199]
*miR-596*	Endometrial	DM_hyper_		[196]
*miR-615*	Pancreatic ductal adenocarcinoma	DM_hypo_	IGF2	[60,131]
*miR-618*	Endometrial	DM_hyper_		[196]
*miR-874*	Breast	DM_hyper_		[213]
*miR-941*	Colorectal cells	DM_hyper_		[214,215]
*miR-1224*	Bladder	DM_hyper_		[214,216]
*miR-1237*	Colorectal cells	DM_hyper_		[214]
*miR-1247*	Colorectal and gastric cells, pancreatic, non-small cell lung	DM_hyper_	RARA, STX1B, RCC2	[62,214,215,217]
*Let-7a*	Ovarian, acute myeloid leukemia, lung, nasopharyngeal carcinoma cells	DM_hyper_, DM_hypo_	C-MYC	[218,219,220,221]
*Let-7c*	Hepatocellular	HMT		[118]

DM_hyper_: DNA hyper-methylation; DM_hypo_: DNA hypo-methylation; HMT: histone methyl-transferase; HDA: histone de-acetilase; HAT: histone acetyl-trasferase. Targets are referred to epigenetically modified miRNAs.

**Table 2:** MicroRNAs target epigenetic complex at post-transcriptional level.

MicroRNAs	Target	Cancer Type	Reference
*miR-15a/16-1*	BMI	Ovarian	[244]
*miR-26a*	EZH2	Burkit lymphoma	[238]
*miR-29a/b*	DNMT3A-B, DNMT1	Lung, acute myeloid leukemia, hepatocellular	[31,32,228]
*miR-30a*	HP1γ	Colorectal	[255]
*miR-101*	EZH2	Prostate, bladder transitional cell carcinoma	[239,240]
*miR-128*	BMI, SUZ12	Glioma	[235,243]
*miR-137*	EZH2	Cervical	[256]
*miR-140*	DNMT1, HDAC4	Hepatocellular, osteosarcoma, colorectal	[257,258]
*miR-143*	DNMT3A	Colorectal	[259]
*miR-145*	HDAC2	Hepatocellular	[260]
*miR-148*	DNMT3B	Cervical cancer cells	[229]
*miR-148a*	DNMT1	Cholangiocarcinoma, gastric	[34,261]
*miR-152*	DNMT1	Cholangioarcinoma, breast	[34,181]
*miR-155*	HDAC4	B-cells lymphoma	[252]
*miR-185*	DNMT1	Glioma	[262]
*miR-194*	BMI	Endometrial	[245]
*miR-200b*	SUZ12, BMI	Breast, hepatocellular	[192,234]
*miR-200c*	BMI	Breast	[263]
*miR-203*	BMI	Multiple myeloma	[247]
*miR-218*	BMI	Glioma	[246]
*miR-221*	HDAC6	Liver	[264]
*miR-449a*	HDAC1	Prostate	[250]
*miR-K12-4-5p*	RBL2	Kaposi’s sarcoma-associated herpesvirus	[33]

**Table 3:** MicroRNAs acting as transcriptional regulator.

MicroRNA	Target	TGS/TGA	Reference
*miR-10a*	*HOXD4*	TGS	[269]
*miR-205*	*IL24*	TGA	[270]
*miR-205*	*IL32*	TGA	[270]
*miR-223*	*NFI-A*	TGS	[271]
*miR-320*	*POLR3D*	TGS	[272]
*miR-373*	*CDH1*	TGA	[273]
*miR-373*	*CSDC2*	TGA	[273]
*miR-423* (*synthetic*)	*PR*	TGS	[274]
*miR-483*	*IGF2*	TGA	[275]
*miR-589*	*COX2*	TGA	[260]
*miR-774*	*Cnnb1*	TGA	[276]
*miR-1186*	*Cnnb1*	TGA	[276]
